# The D-dimer level predicts the prognosis in patients with lung cancer: a systematic review and meta-analysis

**DOI:** 10.1186/s13019-021-01618-4

**Published:** 2021-08-28

**Authors:** Mingsheng Ma, Run Cao, Wei Wang, Biying Wang, Yichen Yang, Yunchao Huang, Guangqiang Zhao, Lianhua Ye

**Affiliations:** 1 Department of Thoracic Surgery, The Third Affiliated Hospital of Kunming Medical University, No. 519 Kunzhou Road, Xishan District, Kunming City, Yunnan Province China; 2 Department of Cardiothoracic Surgery, The Sixth Affiliated Hospital of Kunming Medical University, Yuxi, China

**Keywords:** D-dimer, Prognosis, Lung cancer

## Abstract

**Objective:**

Although the significance of increased plasma D-dimer levels in activating coagulation and fibrinolysis has been reported, it is still controversial whether it can be used to predict the prognosis of lung cancer patients. This meta-analysis was performed to explore the beneficial role of plasma D-dimer as a prognostic factor in lung cancer patients according to a larger sample capacity.

**Materials and methods:**

MEDLINE, EMBASE, and Cochrane Central databases were searched from inception to January 2021. The data are mainly hazard ratio(HR) with 95% confidence interval (CI) and Kaplan–Meier survival curves. The publication bias was examined by Egger’s test.

**Results:**

Finally, a total of 28 studies, enrolling 8452 patients were included in the current meta-analysis. Our results showed that the OS (HR = 1.742, 95%CI:1.542–1.969, *P* < 0.001) and PFS (HR = 1.385, 95%CI:1.169–1.641, *P* = 0.003) in the high D-dimer group were significantly lower than those in the low D-dimer group. Subgroup analysis suggested that localization, detection methods and disease stage had an important effect on the prognosis.

**Conclusion:**

This meta-analysis revealed that the high plasma D-dimer level leads to lower survival than in the low D-dimer level, which might provide an important clue for high plasma D-dimer level as an independent factor of poor prognosis in patients with lung cancer.

**Supplementary Information:**

The online version contains supplementary material available at 10.1186/s13019-021-01618-4.

## Introduction

Lung cancer has become the most common malignant tumor and the leading cause of cancer death in the world. Activation of coagulation and fibrinolysis is usually associated with most malignant tumors [1], although the exact molecular mechanism remains incompletely understood. Previous studies have found that malignancy can affect the hemostatic system; however, the activation of the hemostatic system can influence the biological behavior of tumors [2]. The coagulation and fibrinolysis system activation can accelerate the growth and invasion of tumor cells, thus affecting cancer progression [3]. Lung cancer patients reveal an abnormal coagulation state, including venous thromboembolism (VTE) [4]. Tumor-induced thrombosis has a remarkable effect on the prognosis of patients with cancer.

Plasma D-dimer is the lysis end-product of cross-linked fibrin protein degradation and is a critical forecast indicator of coagulation dysfunction [5]. Plasma D-dimer levels increase by promoting fibrin formation and fibrinolysis. In the past few years, plasma D-dimer elevated levels have been attracted much attention with different malignant tumors, including colorectal cancer, gastric colorectal, cervical, breast, esophagus cancer [6, 7]. Plasma D-dimer levels have played a vital role in excluding thrombosis associated with clinical cancer [8]. In these studies, a high D-dimer level was found and associated with the prognosis of the patients. Therefore, the plasma D-dimer level can be used as an effective prognostic predictor.

D-dimer elevated levels have also been found in lung cancer patients and associated with a poor prognosis [9, 10]. It has been reported that tumors with a higher angiogenesis and metastasis manner biologically degree behave related to activating the coagulation system [11]. However, these analyzed studies are only based on data such as certain quantities of small cell lung cancer (SCLC) patients and tumor staging (I–IV). The prognostic value of D-dimer in lung cancer remains limited. Although many studies demonstrated that a high D-dimer level is related to the prognosis of lung cancer, the plasma D-dimer levels as a lung cancer prognostic criterion are still controversial.

In this study, we performed a meta-analysis of collecting current comparative data to further determine D-dimer level's prognostic significance in patients with lung cancer.

## Methods

### Search strategy and eligibility criteria

We performed a literature search through the following databases: Pubmed, Cochrane Central databases, Web of Science and EMBASE databases for studies published before January 2021. The keywords we defined were as follows: “lung cancer,” “D-dimer,” and “prognosis” and the search strategy were used: (“lung cancer” OR “Pulmonary Neoplasm” OR “Lung Neoplasm”) AND (“D-dimer” OR “fibrinolysis”) AND (“prognosis” OR “prognostic”). The references of relevant studies and review articles were also checked to identify additional studies. Two authors (MM and WW) assessed the titles and abstracts independently to extract the full articles for all potentially relevant studies from each eligible report. This systematic review with individual patient data meta-analysis was registered on INPLASY (INPLASY202170096).

All retrieved articles included observational studies assessing the inclusion and exclusion criteria described. The selected studies inclusion criteria were as follows: 1) patients with lung cancer including any treatments; 2) articles investigating the correlation of D-dimer levels which collected before treating with the survival of lung cancer; 3) overall survival (OS) and relapse-free survival (PFS); 4) data: the necessary survival data must be provided, including hazard ratio (HR), 95% confidence interval (CI) and Kaplan–Meier curve; 5) full text is available. The following criteria were excluded: 1) records were not written in English; 2) records such as abstracts, letters, reviews, case reports, or nonclinical studies; 3) full text is unavailable; and 4) follow-up time less than 5 years.

### Data extraction and quality assessment

The following data were performed by the two authors (BW and YY) independently. The information should be extracted included:(1)author name, publication year, sample size, age, gender, median follow-up time; (2)disease stage, treatment approach, histology type, detection method, location; (3) the risk ratio (HR) and their associated 95% CI, The primary prognosis outcomes were OS and PFS. If the title and abstract cannot be classified, the full text should be read. Two authors (YH and GZ) evaluated the quality of studies independently if disagreement occurred, and the third investigator made the final agreed decision (LY). The value was assessed through methods in the literature if the HR was not given directly [12, 13]. The quality of the studies was evaluated in accordance with the Newcastle–Ottawa Scale (NOS). NOS contained three domains: patient selection (0–4 points), comparability (0–2 points), and outcome (0–3 points). NOS scores ranged from 0 to 9 points, and studies with an NOS score ≥ 6 were considered to be of high quality.

### Statistical analysis

The association of D-dimer with OS and PFS was evaluated by pooling HRs and 95% CIs. Heterogeneity was assessed with Chi-square and the I^2^ index. A P-value ≥ 0.1 and I^2^ < 50% were considered not statistically significant, and the fixed effects model was used. A P-value < 0.1 and I^2^ ≥ 50% was regarded as high heterogeneity. The random-effected model was chosen to pool the heterogeneous studies [14]. Publication bias was used to assess by Begg’s funnel plot. We used STATA version 15.1 to perform the meta-analysis. All statistical tests were two-tailed, and *P* < 0.05 was set statistically significant.

## Results

### Study and patient characteristics

**Fig. 1:**
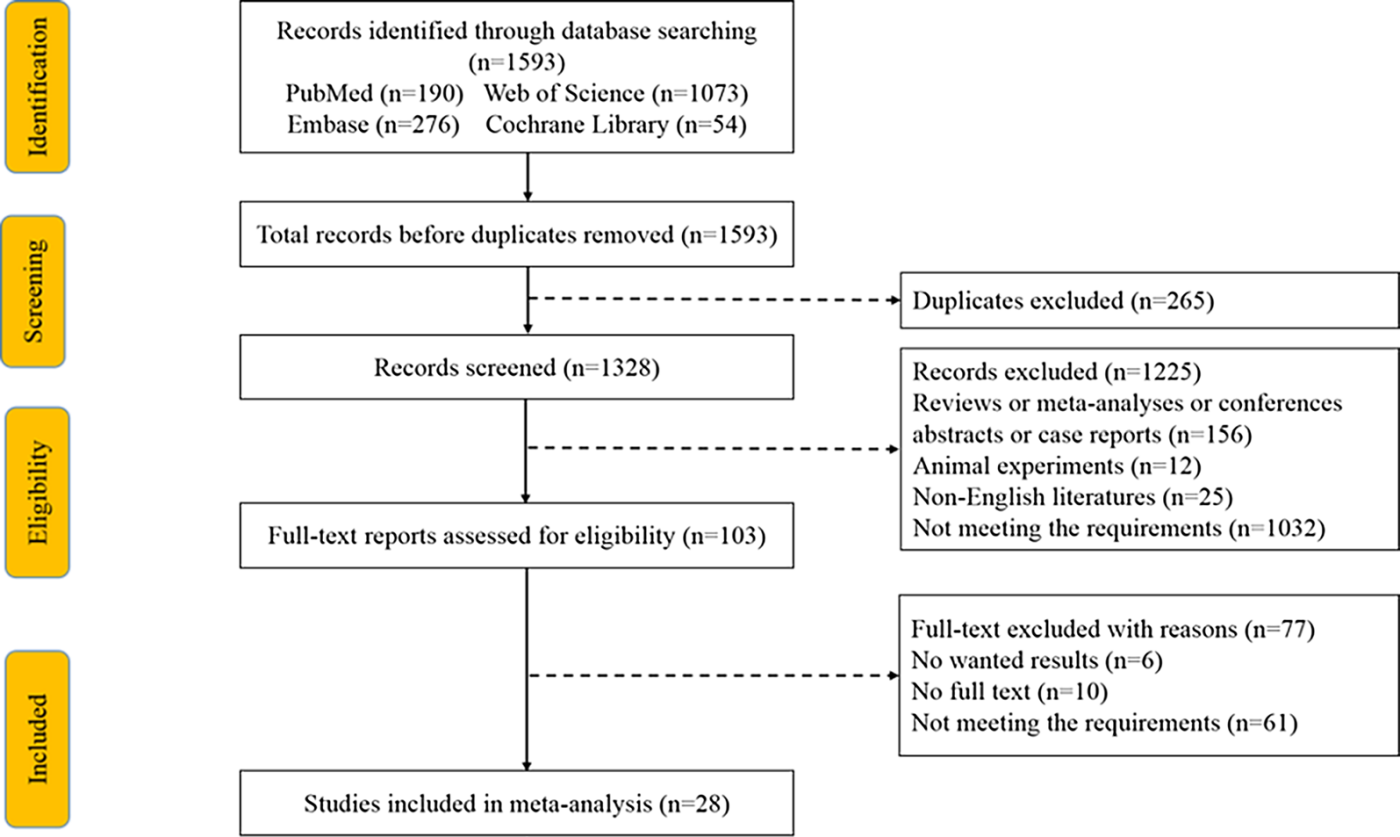
The flow diagram of literature search

We screened 1593 studies from the searches. After carefully testing these articles, a total of 28 studies, including 8452 patients published between 1997 and 2021, were included in the meta-analysis. In this analysis, the high D-dimer group was chosen as the reference. All included studies were retrospective. The flow diagram of the detailed process of study selection is shown in Fig. 1. Among them, 4 studies were from Japan, 15 studies were performed in China, 3 studies were from Italy, 5 studies were from Turkey, 1 study was from Australia, Austria, and Russia, 27 studies used OS as a unique prognostic indicator, 7 studies described PFS. The characteristics of the enrolled studies are summarized in Table 1. All patients included had a stage I-IV disease and received different treatments. The median age of these patients was 57 to 62 years old. All but five studies were followed up for a relatively long time. However, some studies have different cut-off values. The HR and 95%CI were obtained directly or indirectly through multivariate Cox regression analysis.

**Table 1:** Summary table of the meta-analysis

Auther(year)	Country	Samplesize(N) (F/M)	Cutoff value	Age (range)	Follow-up (median)	Detection method	Outcomes	Quality assessment
Buccheri et al. [17]	Italy	286(31/255)	0.5 g/mL	61 (32–87)	34 months	Latex assay	OS	7
Taguchi et al. [18]	Japan	70	150 ng/ml	65 (20–83)	15 months	ELISA	OS	5
Ferrigno et al. [19]	Italy	343(31/304)	0.5 ug/ml	68 (39–86)	-	Immunoturbidimetry	OS	8
Pavey et al. [20]	Australia	166(38/128)	32.99 ng/ml	64 (30–86)	270 days	ELISA	OS	8
Buccheri et al. [21]	Italy	826(99/727)	0.5 ug/ml	67 (35–89)	34 weeks	Immunoturbidimetry	OS	8
Altiay et al. [23]	Turkey	78(5/73)	0.65 ug/ml	61 (37–82)	264 days	ELISA	OS	8
Komurcuoglu et al. [24]	Turkey	100(14/86)	1250 ng/dl	67	-	ELISA	OS	6
Masago et al. [25]	Japan	99(28/71)	0.6 ng/ml	72(35–88)	0–800 days	ELISA	OS	8
Ay et al. [26]	Austria	182	0.84 g/mL	62 (52–68)	731 days	Latex assay	OS	7
Tas et al. [2]	Turkey	110(10/100)	360 IU/ml	59(35–80)	20.3 weeks	Microparticle Enzyme Immunoassay	OS	7
Zhang et al. [27]	China	232 (83/149)	0.3 ug/ml	61(30–86)	47.0 months	Immunoturbidimetry	OS	6
Fukumoto et al. [28]	Japan	237(85/152)	0.50 lg/ml	69(31–85)	51.6 months	-	OS	7
Ursavaş et al. [29]	Turkey	65 (10/55)	375 μg/L	60	0–1,000 days	Latex assay	OS	
Ge et al. [30]	China	82(27/55)	-	64 (44–72)	-	Immunoturbidimetry	OS/PFS	8
Inal et al. [9]	Turkey	72(16/56)	1900 ng/mL	-	574.14 days	ELISA	OS	8
Wang et al. [31]	China	1929(604/1325)	0.5 μg/mL	-	18.0 months	Immunoturbidimetry	OS/PFS	7
Chen et al. [22]	China	393(71/322)	0.5 ug/ml	57(49–65)	12 months	Immunoturbidimetry	OS/PFS	6
Zhu et al. [32]	China	74(17/57)	0.55 ug/L	57(42–80)	11.5 months	Immunoturbidimetry	OS/PFS	8
Jiang [33]	China	107(23/84)	0.55 mg/L	63.0(58.5,68.0)	9 months	Immunoturbidimetry	OS	8
Sun [18]	China	272(109/163)	0.55 mg/L	65	-	-	OS	6
Zhang et al. [34]	China	160(31/129)	500 ng/ml	59 (23–83)	-	Immunoturbidimetry	PFS	8
Fan et al. [35]	China	82(15/67)	0.55 mg/L	60(28–82)	0–50 months	Immunoturbidimetry	PFS/OS	8
Hou et al. [36]	China	395(170/225)	0.20 mg/L	64(56–69)	13.2 months	Immunoturbidimetry	OS	6
Liang et al. [37]	China	456(138/318)	500 ng/ml	61(35–81)	42 months	Immunoturbidimetry	OS	8
Shiina et al. [38]	Japan	235(89/146)	1.0 μg/ml	70	0–3 years	-	OS	8
Chen et al. [15]	China	233(70/1630)	500 mg/ml	67	0–60 months	ELISA	OS	7
Liu et al. [16]	China	651(216/435)	0.5 mg/L	60	0–80 months	Immunoturbidimetry	OS	7
Moik et al. [39]	Russia	277(103/174)	1 mg/dl	61(56–67)	24 months	Latex assay	OS/PFS	8

Auther(year)	Treatment					Histology type	Disease stage (T0/T1/T2/T3/T4)
	Chemotherapy (alone/combination)	Radiotherapy	Chemo-radiation	Surgery	No anticancer treatment or supportive care		
Buccheri et al. [17]	-	-		-	-	E/S/A/L/U 112/37/67/18/52	I/II/III/IV 42/31/119/94
Taguchi et al. [18]	50	20	0	NSCLC 49; SCLC 21	-		
Ferrigno et al. [19]	175	8	-	66	26%	E/A/S/L/U 125/80/34/16/88	I/II/III/IV 69/26/124/121
Pavey et al. [20]	-	-	-	-	-	NSCLC 166(A/E/L/O 68/76/13/9)	-
Buccheri et al. [21]						E/S/A/L/U 296/93/210/48/179	I/II/III/IV /149/57/310/305
Altiay et al. [23]	74	57		-	4	NSCLC 60; SCLC 18	III/IV 35/43
Komurcuoglu et al. [24]	-	-		-	-	NSCLC 87; SCLC 13	II/III/IV 15/50/35
Masago et al. [25]	67	2	8	-	22	A/E/N 68/18/13	III/IV42/57
Ay et al. [26]	-	-	-	-	-	-	-
Tas et al. [2]						E/A/U/S 26/30/28/16	
Zhang et al. [27]	-	-	-	232	-	E/A 111/121	I-II/III 173/59
Fukumoto et al. [28]	-	-	-	237	-	E/A/L/O 51/162/7/17	-
Ursavaş et al. [29]	-	-	-	-	-	E/A/S/U 32/11/17/5	I/II/III/IV 20/5/16/32
Ge et al. [30]	82	-	-	-	-	N-SCC/SCC 53/29	IIIB/IV 10/72
Inal et al. [9]	72	-	-	-	-	E/A/N/S 19/14/24/15	III/IV 33/22
Wang et al. [31]	-	-	-	-	-	A/O 1046/885	IV1929
Chen et al. [22]	126	-	267	-	-	SCLC 393	-
Zhu et al. [32]	74	-	-	-	-	SCLC 74	-
Jiang [33]	25	-	82	-	-	SCLC 107	-
Sun [18]	177	-	-	-	-	N-SCLC 272	IV 272
Zhang et al. [34]	160	-	-	-	-	SCLC 160	LD/ED 38/122
Fan et al. [35]	27	-	51	-	-	SCLC 82	I + II + IIIa/IIIb + IV 19/63
Hou et al. [36]	-	-	-	395	-	NSCLC(E/A/O) 53/329/13	I-II/III 341/54
Liang et al. [37]	-	-	-	456	-	NSCLC 61	I/II/III 170/101/185
Shiina et al. [38]	46	2	9	235	-	Adenocarcinoma/Squamous cell carcinoma/LCNEC/Large cell carcinoma/Pleomorphic carcinoma/Carcinoid 186/55/2/2/2/2 A/E/L/L/P/C 186/55/2/2/2/2	-
Chen et al. [15]	-	-	-	-	-	E/A/O 55/124/54	III/IV 110/123
Liu et al. [16]	492	70	89	E/A/O 126/504/21	III/IV98/553		
Moik et al. yy[39]	277	277	-	277	-	NSCLC/SCLC 231/46	I/II/III/IV 1/4/76/196

S:SCLC/small cell lung cancer;N:N-SCLC/non-small cell lung cancer;E:Epidermoid/Squamous cell carcinomas

A: Adenocarcinoma;U: unclassified;L: Large cell carcinoma;O: others; LCNEC: large cell neuroendocrine carcinoma;'-': not mentioned

### Quality assessment and risk of bias

All studies were retrospective cohort studies, and we evaluated the quality based on the modified NOS scale. The results of the quality assessment were listed in Table 1. All studies are considered to be of high quality, indicating that the risk of bias in our entire study is very low.

### Meta-analysis of the D-dimer level on OS and PFS with lung cancer patients

**Fig. 2:**
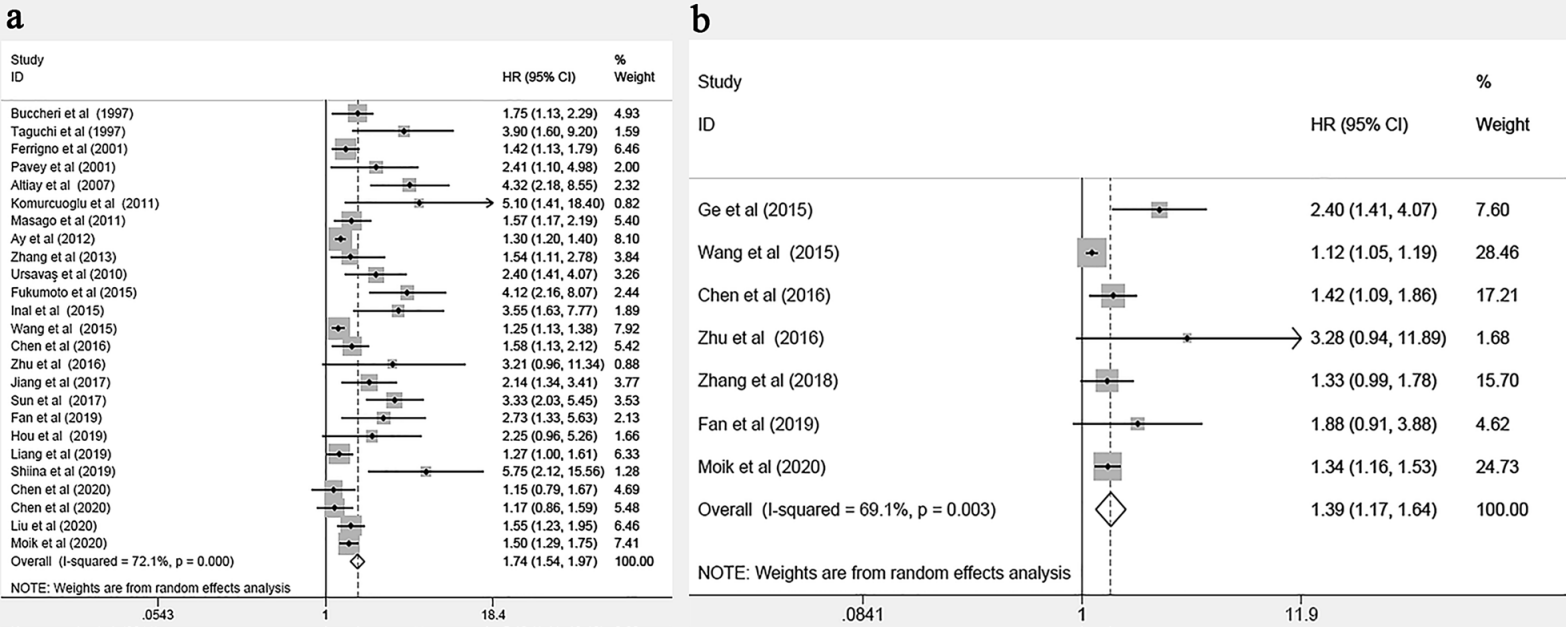
Estimated HR summary for **a** OS in patients, **b** PFS in patients

Twenty-seven studies included 5,455 patients compared OS between lung cancer patients with high D-dimer level and these with normal D-dimer level. Our meta-analysis found that the OS of lung cancer patients with high D-dimer levels was worse than that of lung cancer patients with normal D-dimer level (random effects: HR = 1.742, 95% CI:1.542–1.969; *P* < 0.001, I^2^ = 72.1%) (Fig. 2a). Seven studies included 2997 patients compared PFS between patients with high D-dimer level and those with normal D-dimer level. Our meta-analysis found that lung cancer patients with high D-dimer level also had a significantly worse PFS (random effects: HR = 1.385, 95% CI:1.169–1.641; *P* = 0.003, I^2^ = 69.1%) (Fig. 2b).

### Subgroup analysis

#### Detection methods

The detection methods used in the studies include immunoturbidimetry assay [15–27], latex assay [1, 28–30], and enzyme-linked immunosorbent assay (ELISA) [9, 31–36]. Since different detection methods may affect the level of D-dimer, we conducted a subgroup analysis to further analyze the impact of different detection methods on the prognosis. The HRs and 95%CI for OS in immunoturbidimetry group assay was 1.363 (1.264–1.470), in group latex assay were 1.513 (1.266–1.810), and in the group ELISA was 2.144(1.466–3.134) ( Additional file 1: Figure S1 a-c). The HR and 95% CI for PFS in immunoturbidimetry groups assay was 1.461 (1.148–1.861), in group latex assay were 1.340 (1.167–1.539) (Additional file 2: Figure S2 a-b).

#### Histological type

95% CI we also divided the studies into histological types (adenocarcinoma / total > 25% or < 25%). We found that the HR and 95%CI of OS in > 25% adenocarcinoma group were 1.639 (1.298–2.069), and 1.976 (1.651- 2.364) for < 25% adenocarcinoma (Additional file 3: Figure S3a-b). In the adenocarcinoma > 25% group, the HR and 95%CI of PFS was 1.120(1.052,1.192), and those of adenocarcinoma < 25% were 1.461 (1.148–1.861) (Additional file 4: Figure S4 a-c).

#### Disease stage

We also divided the studies into clinical stage (stage III–IV/total > 80% or < 80%). The HRs and 95%CI for OS in group stage III–IV/total > 80% was 1.681(1.377–2.053), in group stage III–IV/total < 80% was 1.352 (1.265–1.445) Additional file 5: Figure S5 a-c. The HRs and 95% CI for PFS in group stage III–IV/total > 80% was 1.431(1.022–2.004), in group stage III–IV/total < 80% was 2.400 (1.413–4.078) (Additional file 6: Figure S6 a-c).

#### Other groups

Three reports originated from Europe, Twenty from Asia, and two from Oceania. When we subgrouped the analysis by patients’ resources, the HRs and 95% CI for OS in patients in Asia 1.979 (1.646–2.39), Europe were 1.504(1.335–1.695) and Oceania were 1.572 (0.899–2.746) (Additional file 7: Figure S7 a-c), respectively. The HRs and 95% CI for PFS in patients in Asia 1.461 (1.148, 1.861, Europe were 1.340(1.167–1.539) (Additional file 8: Figure S8 a-b). We also divided the studies into different treatments (non-surgical, surgical, or mixed and found that the HRs and 95% CI for OS in the non-surgical group were 2.380 (1.780–3.0) the surgical group was 1.910 (1.17–3.14) and for and mixed group 1.688 (1.333–2.139) (Additional file 9: Figure S9 a-b). The HRs and 95% CI for PFS in the non-surgical group were 310 (1.076–1.596) and mixed group 2.400 (1.413–4.078) (Additional file 10: Figure S10 a-b).

The results of meta-analyses of prediction value for overall and subgroup analysis were shown in Table 2.

**Table 2:** results of meta-analyses of prediction value

Survival outcome	No	HR [95% CI]	Log-rank p	Heterogeneity (p, I2(%))	Publication bias
OS	27	1.742(1.542,1.969)	< 0.001	< 0.001,72.1%	0.871
location					
Asia	20	1.979(1.646,2.379)	< 0.001	< 0.001,75.3%	
Europe	3	1.504(1.335,1.695)	< 0.001	0.623,0.0%	
Oceania	2	1.572(0.899,2.746)	0.112	0.111,60.6	
Histology type (adenocarcinoma/total > 25%)					
Yes	8	1.639(1.298,2.069)	< 0.001	< 0.001,71.8%	
No	15	1.976(1.651,2.364)	< 0.001	< 0.001,75.3%	
Tumor stage (III + IV/total > 80%)					
Yes	9	1.681(1.377,2.053)	< 0.001	< 0.001,78.0%	
No	8	1.352(1.265,1.445)	< 0.001	0.065,47.4%	
Detection method					
ELISA	7	2.144(1.466,3.134)	< 0.001	< 0.001,74.4%	
latex assay	4	1.513(1.266,1.810)	< 0.001	0.026,67.6%	
immunoturbidimetry assay	10	1.363(1.264,1.470)	< 0.001	0.065,44.1%	
Surgery					
Non-surgical	8	2.380(1.78,3.18)	< 0.001	0.019,58.2%	
surgical	4	1.910(1.17,3.14)	< 0.001	0.008,74.5%	
mixed	5	1.688(1.333,2.139)	< 0.001	0.020,65.6%	
PFS	7	1.385(1.169,1.641)	< 0.001	0.003,69.1%	
location					
Asia	6	1.461(1.148,1.861)	0.002	0.007,68.5%	
Europe	1	1.340(1.167,1.539)	< 0.001		
Histology type (adenocarcinoma/total > 25%)					
Yes	1	1.340(1.167,1.539)	< 0.001		
No	6	1.461(1.148,1.861)	0.002	0.007,68.5%	
Tumor stage (III + IV/total > 80%)					
Yes	3	1.431(1.022,2.004)	0.037	0.005,81.1%	
No	1	2.400(1.413,4.078)	0.001		
Detection method					
latex assay	1	1.340(1.167,1.539)	< 0.001		
immunoturbidimetry assay	6	1.461(1.148,1.861)	0.002	0.007,68.5%%	
Surgery					
Non-surgical	5	1.310(1.076,1.596)	0.007	0.074,53.0%	
mixed	1	2.400 (1.413,4.078)	0.001		

### Sensitivity analysis and publication bias

**Fig. 3:**
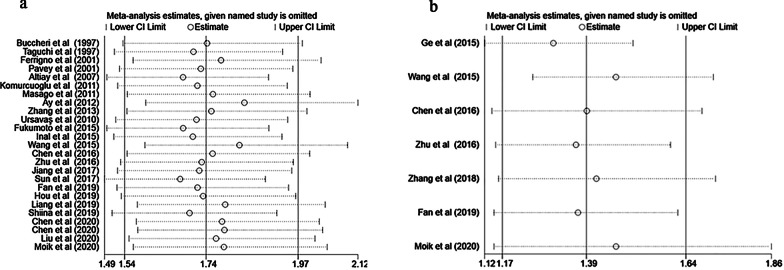
The sensitivity analysis of OS (**a**) and PFS (**b**)

**Fig. 4:**
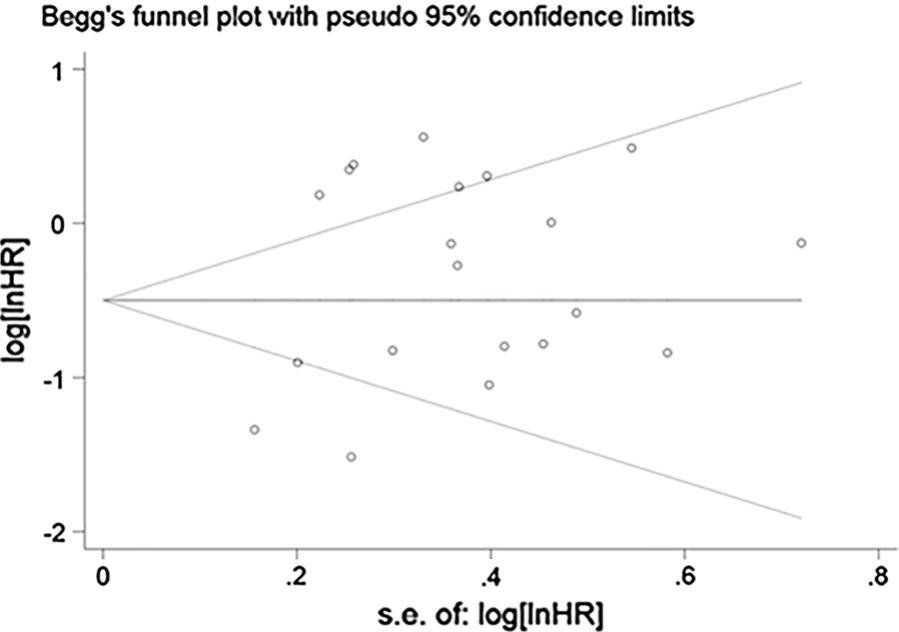
Summary of Begg’s funnel plots of publication bias for OS in all patients

The stability of OS and PFS was evaluated by sensitivity analysis. Sensitivity analysis is to remove each study in turn to observe whether to change the original overall analysis results, the results show that each study deleted in turn did not change the original results, indicating that the results are more reliable (Fig. 3a, 3b). Publication bias was tested by Begg’s test, and the results showed that OS had no publication bias (*P* = 0.871) (Fig. 4).

## Discussion

As we all know, lung cancer has been widely concerned and studied by the society because of its high mortality rate. In recent years, due to the rapid development of surgical technology, various treatments including radiotherapy and chemotherapy have greatly improved the survival of lung cancer patients. However, we believe that to improve the survival rate and quality of life of lung cancer patients, it is necessary to find a way to predict prognostic survival as soon as possible. Therefore, many researchers are looking for biomarkers that can predict the prognosis of lung cancer, and use this to provide guidance for the later clinical treatment of lung cancer.

D-dimer is involved in the regulation of multiple cancer processes, so in recent years, there have been many studies exploring the relationship between D-dimer levels and the prognosis of lung cancer patients [15, 31]. Han proved that high D-dimer level is associated with the danger of occult tumor in patients with unprovoked venous thromboembolism (VTE) [37]. In cancer patients some experts found that the levels of D-dimer can predict deep vein thrombosis (DVT) [38].These studies found that high level D-dimer may be related to the poor prognosis in tumor patients. Meanwhile, Heit found that tumor cells can promote coagulation system, increase platelet activity and damage vascular endothelial cells [39]. Another research found that activation of the blood coagulation system associated with invasive and migration biological behavior of tumors [40]. In terms of mechanism research, it may be associated with tissue factors, coagulants, colony-stimulating factors, coagulants, inflammatory cytokines, platelet activation markers were produced by tumor cells, These factors can activate the coagulation system and trigger the coagulation cascade through different signaling pathways [41–47]. However, the underlying mechanisms that why elevated D-dimer is related to a poor survival in patients with lung cancer are unknown and need further study.

With regard to clinical significance of D-dimer in lung cancer, whether anticoagulant treatment before cancer- related treatment in lung cancer patients can change their prognosis, and whether D-dimer is associated with clinical staging and tumor tissue type are uncertain. Moreover, the value of D-dimer as prognostic outcomes is unclear, especially in patients undergoing radiotherapy and chemotherapy. Therefore, our meta-analysis needs to approve the prognostic value of D-dimer for lung cancer.

Our meta-analysis investigated the relationship between plasma D-dimer and prognosis in lung cancer patients. The results revealed that D-dimer as an effective coagulation factor has the potential to be a promising biomarker to predict prognosis in lung cancer patients, which could promote doctors’ anticoagulant therapy selection. In our study, we estimated the significance of plasma D-dimer levels in lung cancers. Compared with these patients who have a normal plasma D-dimer, patients with a high level of plasma D-dimer have a worse overall survival prognosis (HR = 1.742, 95% CI:1.542–1.969; *P* < 0.001, I^2^ = 72.1%). We also found that elevated plasma D-dimer is a risk factor of PFS in lung cancers according to pooled HR (HR = 1.385, 95% CI:1.169–1.641; *P* = 0.003, I^2^ = 69.1%). Hence, we think that this meta-analysis can confirm the prognostic significance of plasma D-dimer level in lung tumors.

We concentrated on subgroup analysis of detection method revealed that studies based on the group of ELISA. We found that the HR ratio in the ELISA group was higher than that in the other two groups. In recent years, ELISA, latex method and immune turbidimetry are mainly used to detect D-dimer. However, compared with the other two methods, ELISA has higher sensitivity and specificity, so many hospitals regard ELISA as the gold standard for the detection of D-dimer. However, it takes a lot of time to use ELISA detection, so there is an urgent need for new methods to help reduce the diagnosis time. In addition, the immunoturbidimetry assay group had a lower heterogeneity (HR = 1.363, 95% CI:1.264,1.470, I^2^ = 44.1%), which might be the possible sources of heterogeneity.

Though fibrinolytic and coagulation activation systems have been reported to boost tumor growth through different mechanisms, including promoting angiogenesis, suppressing apoptosis of tumor cells, the mechanism association between the plasma D-dimer level and the aggressiveness of lung cancer remains unclear. Shiina et al. found that tumors in the high D-dimer group frequently invaded vessels [48]. Our subgroup analysis about tumor stage (III + IV/total > 80%) finding high D-dimer level had a worse prognosis of OS and PFS, indicating that D-dimer levels are associated with tumor invasion metastasis. Although high D-dimer levels might be lead to vessel injury due to tumor aggressiveness, the connection between the plasma D-dimer level and vessel invasion is unclear, and more detailed research is needed. Many studies believe that HR > 2 indicates a prognostic biomarker in practical setting [49]. When subgroup analysis of OS was performed according to different treatment methods, the OS HR of non-surgical patients was higher than 2. This indicates that D-dimer has a stronger predictive value in non-surgical patients, which is consistent with the following report that progression-free survival was significantly worse in patients with high D-dimer levels than normal levels at different time points before and after chemotherapy [20].

Higher heterogeneity was found in our meta-analysis for OS and PFS of the prognostic role of D-dimer (*P* < 0.001, I^2^ = 72.1%, *P* = 0.003, I^2^ = 69.1%). To remove these biases, subgroup analyses were performed by the different nations, detecting methods, disease stage, histological type, and different treatments. When we grouped the analysis into III + IV/total < 80% groups, immunoturbidimetry assay group, and Europe group, the heterogeneity for OS could shrink to 47.4%,44.1%, and 0. Heterogeneity in other subgroups can be reduced or eliminated accordingly.

Several limitations should be addressed in our meta-analysis. First, our meta-analysis could only include retrospective cohort studies with limited sample size and patient selection bias for analysis. As a result, the statistical power of our meta-analysis could be decreased. Second, the main outcomes could not be fully obtained from all these original studies, and PFS cannot be tested for publication bias due to insufficient data. Moreover, D-dimer levels were measured only once which may result in errors. In conclusion, more prospective research with larger sample size is executed to confirm the further relationship between preoperative D-dimer molecular markers and prognostic characteristics.

## Conclusions

We conducted this meta-analysis revealed that the high plasma D-dimer level leads to lower survival than in the low D-dimer level, which might provide an important clue for high plasma D-dimer level as an independent factor of poor prognosis in patients with lung cancer. In the future, more prospective cohort studies are needed to study and verify.

## Supplementary Information

**Additional file 1** Estimated HR summary for a OS in patients with the detection method of immunoturbidimetry,b OS in patients with the detection method of latex assay,c OS with the detection method of ELISA

**Additional file 2** Estimated HR summary for a PFS in patients with the detection method of immunoturbidimetry,b PFS in patients with the detection method of latex assay

**Additional file 3** Estimated HR summary for a OS in patients with the Histological type of > 25% adenocarcinoma,b OS in patients with the Histological type of < 25% adenocarcinoma

**Additional file 4** Estimated HR summary for a PFS in patients with the Histological type of > 25% adenocarcinoma,b PFS in patients with the Histological type of < 25% adenocarcinoma

**Additional file 5** Estimated HR summary for a OS in patients with the Disease stage of stage III–IV/total >80%,b OS in patients with the Disease stage of stage III–IV/total <80%Estimated HR summary for a OS in patients with the Disease stage of stage III–IV/total >80%,b OS in patients with the Disease stage of stage III–IV/total <80%

**Additional file 6** Estimated HR summary for a PFS in patients with the Disease stage of stage III–IV/total >80%,b PFS in patients with the Disease stage of stage III–IV/total <80%

**Additional file 7** Estimated HR summary for a OS in patients in Asia countries,b OS in patients in Europe countries,c OS in patients in Oceania countries

**Additional file 8** Estimated HR summary for a PFS in patients in Asia countries,b PFS in patients in Europe countries

**Additional file 9** Estimated HR summary for a OS in patients with non-surgical,b OS in patients with surgical,c OS in patients with mixed treatments

**Additional file 10** Estimated HR summary for a PFS in patients with non-surgical,b PFS in patients with mixed treatments

## Data Availability

The datasets generated and analyzed during the current study are available from the corresponding author on reasonable request.

## Abbreviations

VTE: Venous thromboembolism
SCLC: Small cell lung cancer
TNM: Tumor node metastasis
DFS: Disease-free survival
OS: Overall survival
RFS: Relapse-free survival
HR: Hazard ratio
CI: Confidence interval
ELISA: Enzyme-linked immunosorbent assay
