# Supplementary figures and images for: The D-dimer level predicts the prognosis in patients with lung cancer: a systematic review and meta-analysis
Source: J Cardiothorac Surg. 2021 Aug 28;16:243. doi: 10.1186/s13019-021-01618-4 (PMC8399789; doi:10.1186/s13019-021-01618-4)

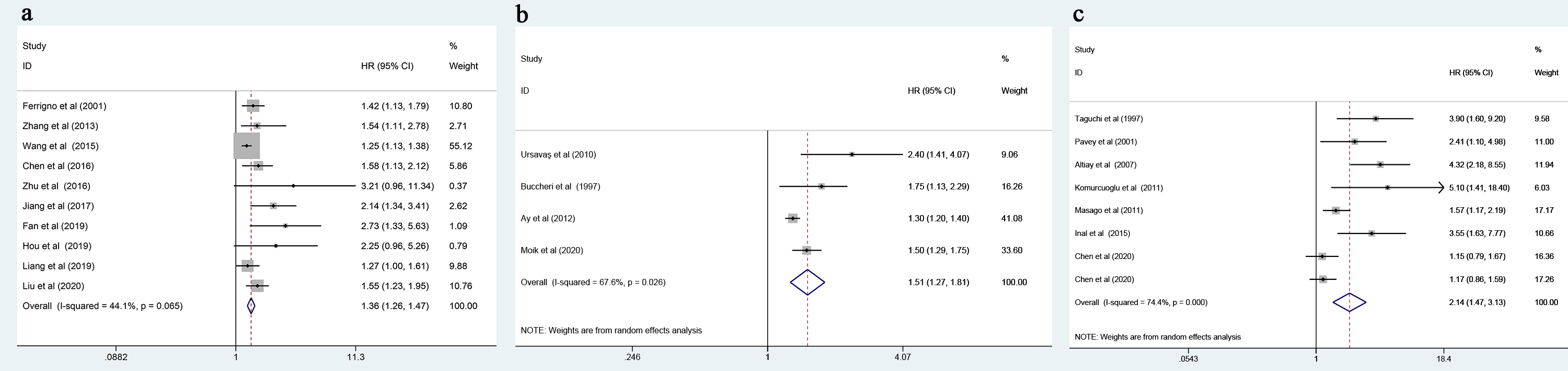

Supplement: Supplementary file 1 — Additional file 1 Estimated HR summary for a OS in patients with the detection method of immunoturbidimetry,b OS in patients with the detection method of latex assay,c OS with the detection method of ELISA [file 13019_2021_1618_MOESM1_ESM.tif]

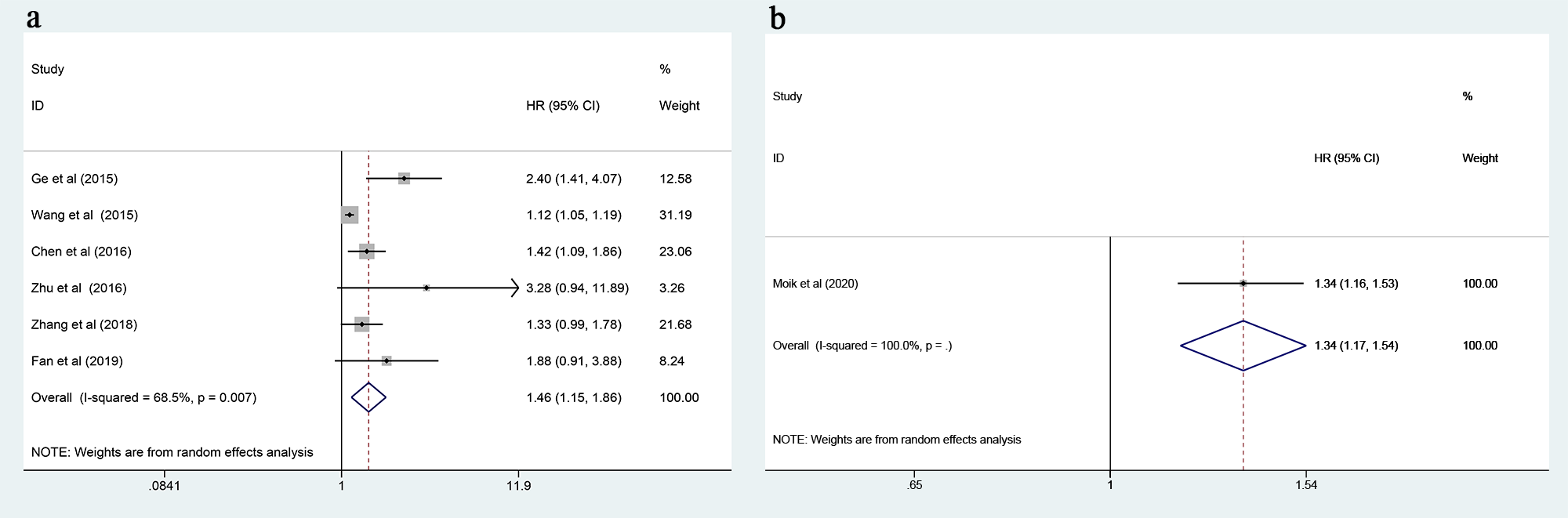

Supplement: Supplementary file 2 — Additional file 2 Estimated HR summary for a PFS in patients with the detection method of immunoturbidimetry,b PFS in patients with the detection method of latex assay [file 13019_2021_1618_MOESM2_ESM.tif]

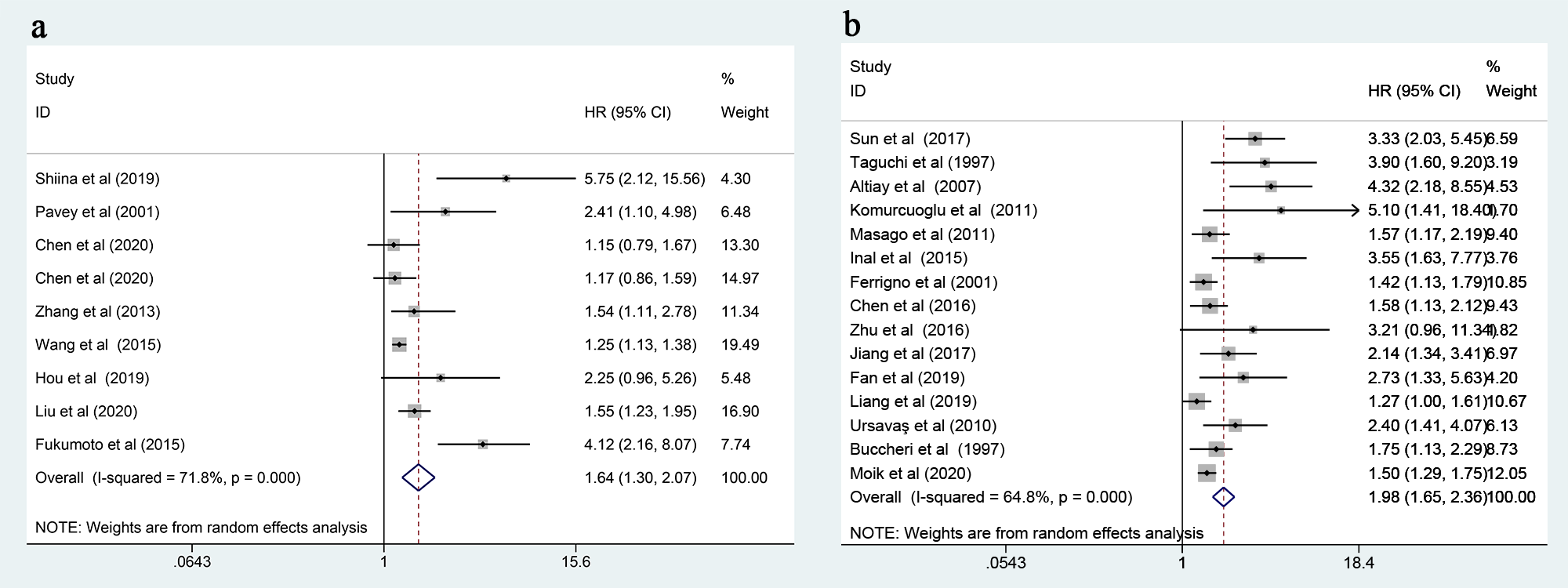

Supplement: Supplementary file 3 — Additional file 3 Estimated HR summary for a OS in patients with the Histological type of > 25% adenocarcinoma,b OS in patients with the Histological type of < 25% adenocarcinoma [file 13019_2021_1618_MOESM3_ESM.tif]

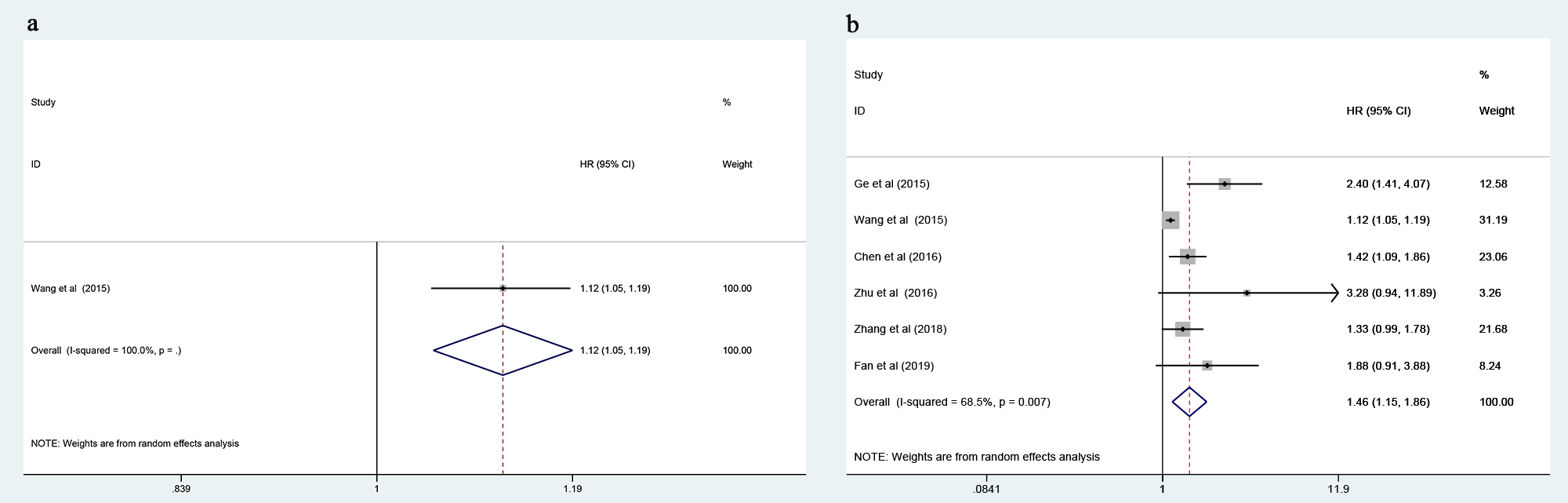

Supplement: Supplementary file 4 — Additional file 4 Estimated HR summary for a PFS in patients with the Histological type of > 25% adenocarcinoma,b PFS in patients with the Histological type of < 25% adenocarcinoma [file 13019_2021_1618_MOESM4_ESM.tif]

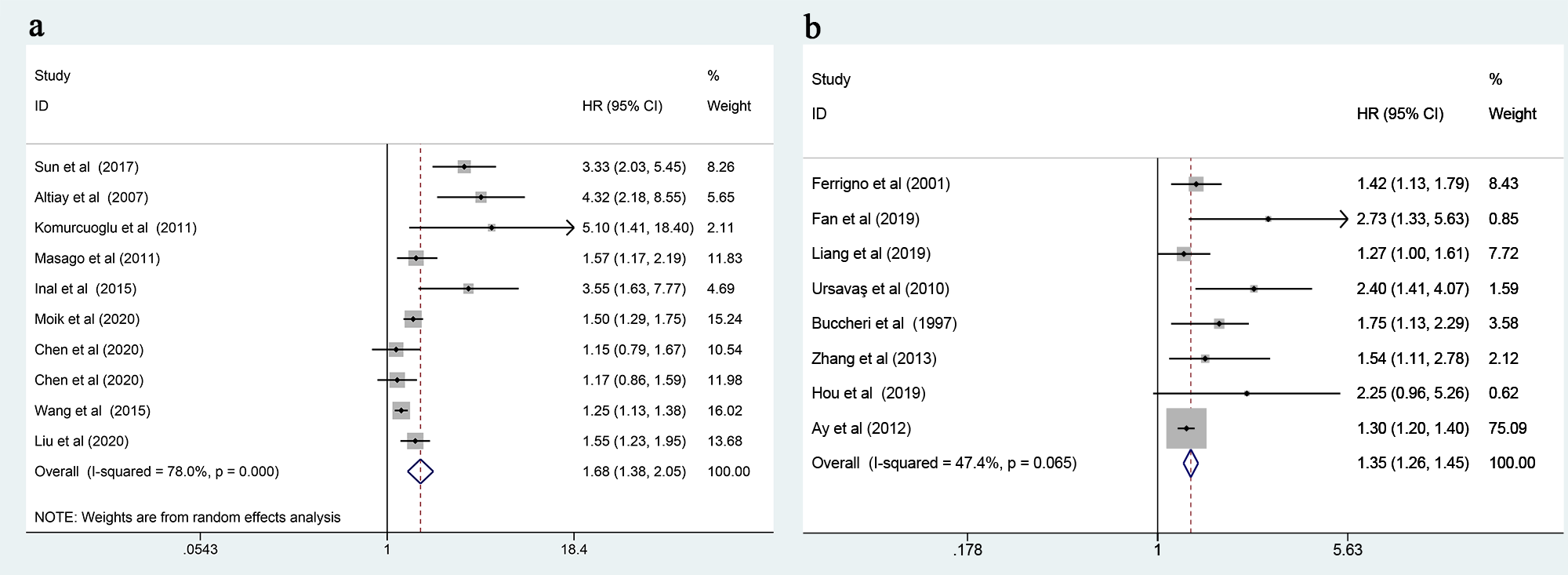

Supplement: Supplementary file 5 — Additional file 5 Estimated HR summary for a OS in patients with the Disease stage of stage III–IV/total >80%,b OS in patients with the Disease stage of stage III–IV/total <80%Estimated HR summary for a OS in patients with the Disease stage of stage III–IV/total >80%,b OS in patients with the Disease stage of stage III–IV/total <80% [file 13019_2021_1618_MOESM5_ESM.tif]

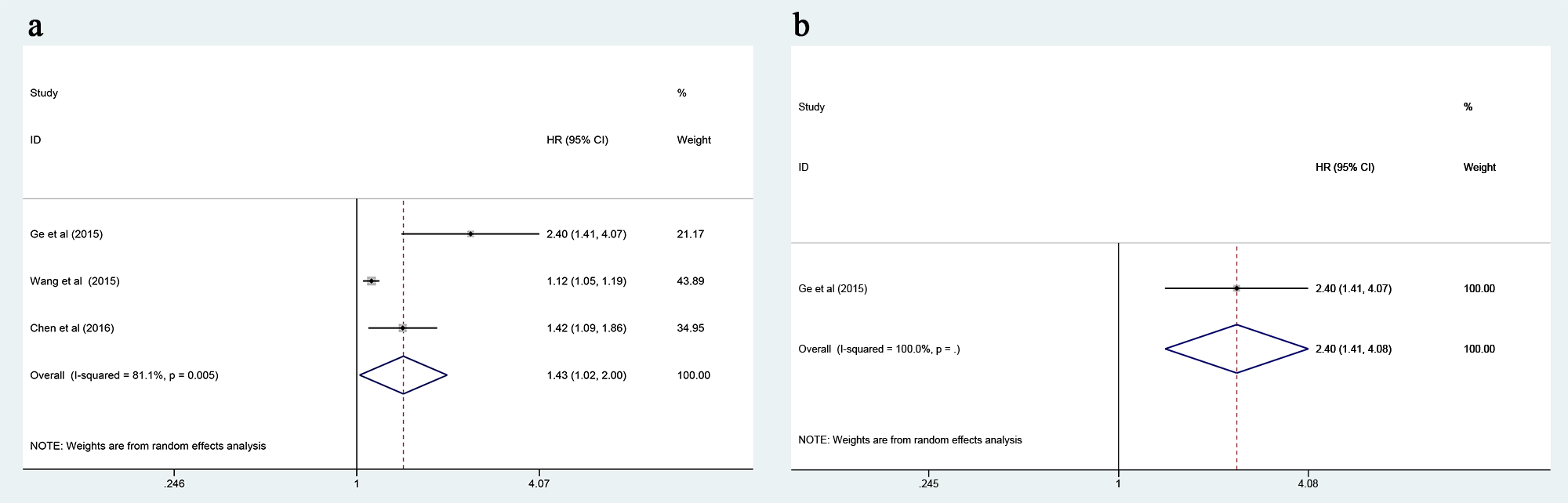

Supplement: Supplementary file 6 — Additional file 6 Estimated HR summary for a PFS in patients with the Disease stage of stage III–IV/total >80%,b PFS in patients with the Disease stage of stage III–IV/total <80% [file 13019_2021_1618_MOESM6_ESM.tif]

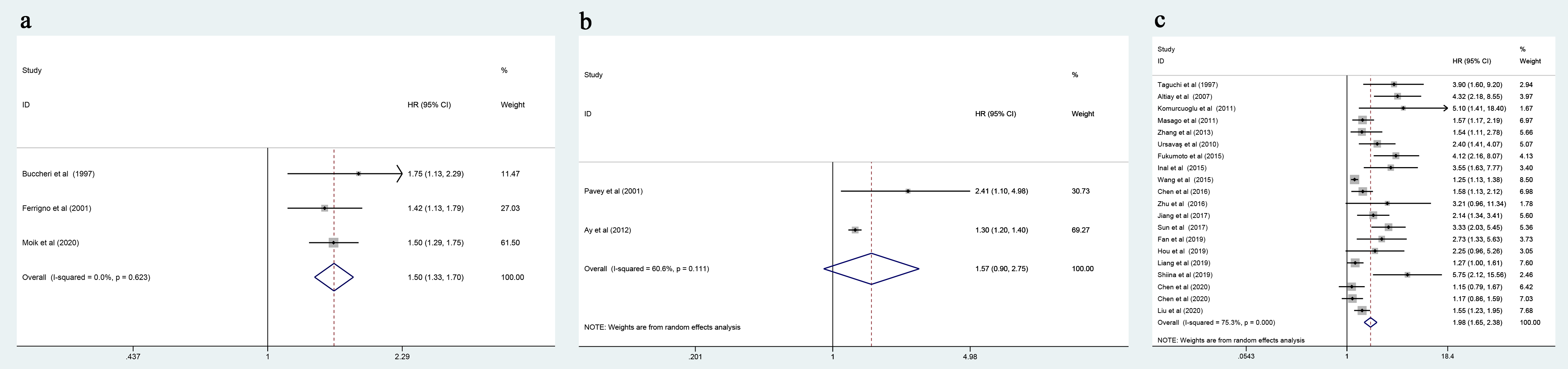

Supplement: Supplementary file 7 — Additional file 7 Estimated HR summary for a OS in patients in Asia countries,b OS in patients in Europe countries,c OS in patients in Oceania countries [file 13019_2021_1618_MOESM7_ESM.tif]

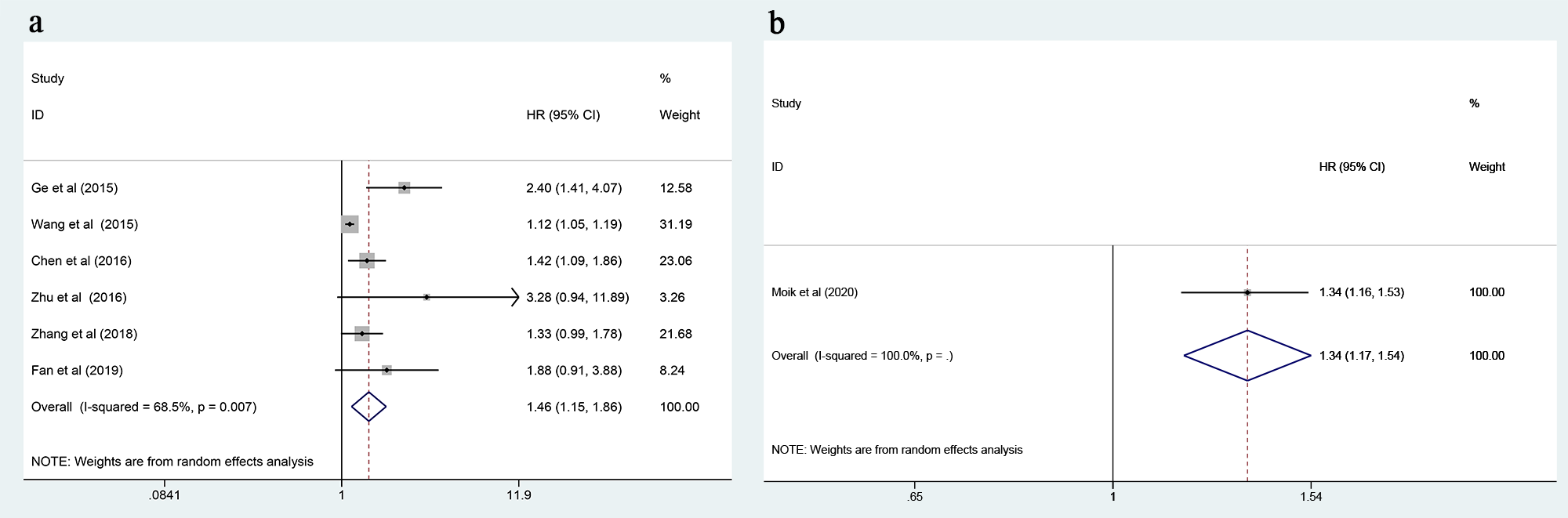

Supplement: Supplementary file 8 — Additional file 8 Estimated HR summary for a PFS in patients in Asia countries,b PFS in patients in Europe countries [file 13019_2021_1618_MOESM8_ESM.tif]

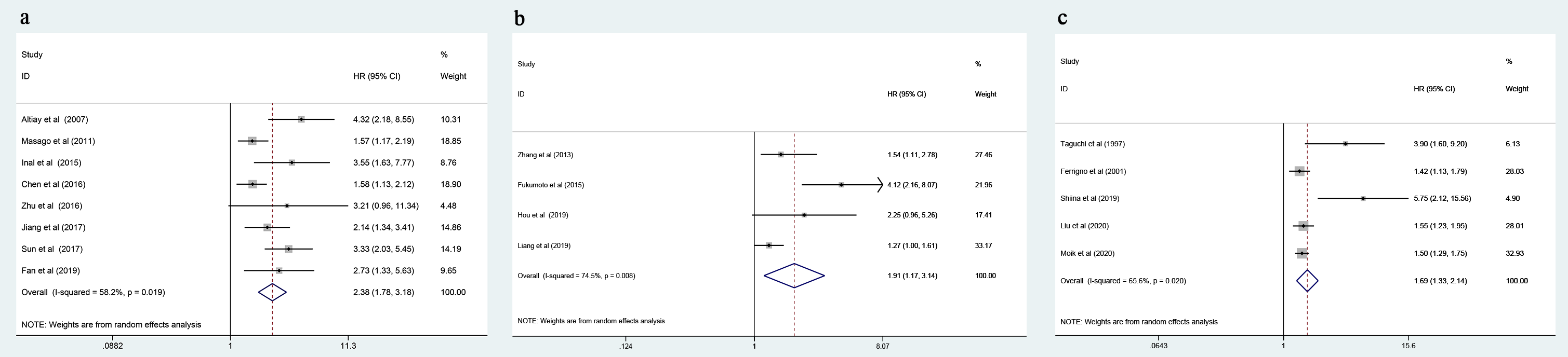

Supplement: Supplementary file 9 — Additional file 9 Estimated HR summary for a OS in patients with non-surgical,b OS in patients with surgical,c OS in patients with mixed treatments [file 13019_2021_1618_MOESM9_ESM.tif]

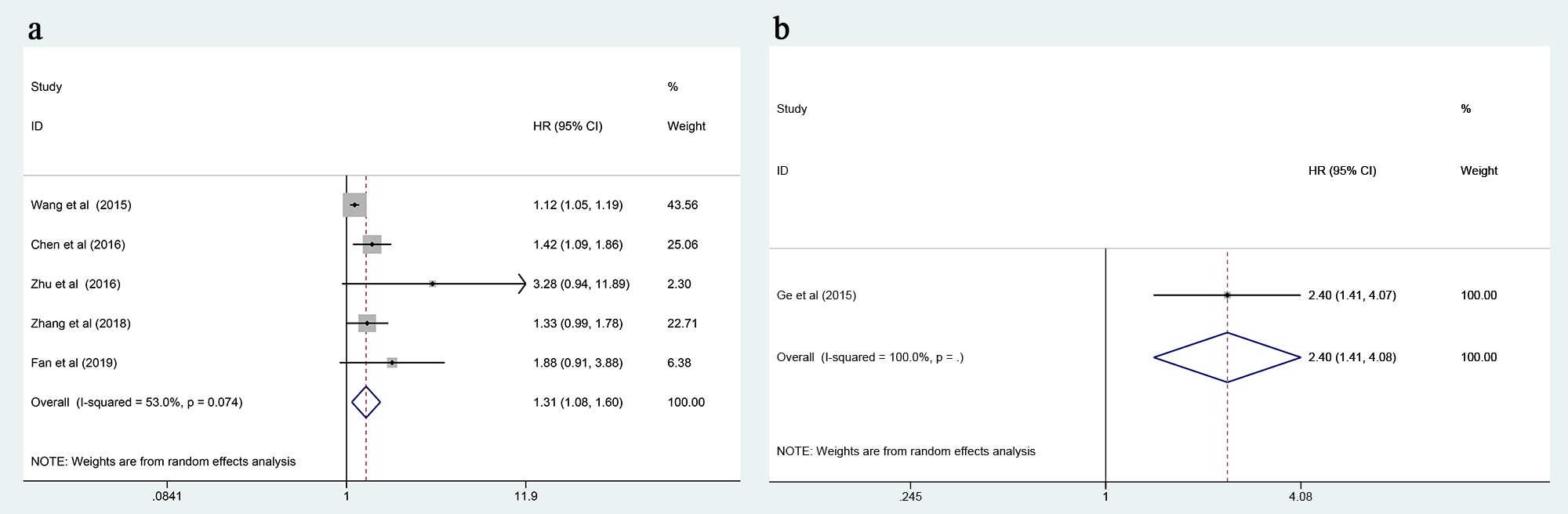

Supplement: Supplementary file 10 — Additional file 10 Estimated HR summary for a PFS in patients with non-surgical,b PFS in patients with mixed treatments [file 13019_2021_1618_MOESM10_ESM.tif]
